# Supplementary material for: Studying phonon coherence with a quantum sensor
Source: Nat Commun. 2024 Jun 11;15:4979. doi: 10.1038/s41467-024-48306-0 (PMC11167028; doi:10.1038/s41467-024-48306-0)
Supplement: Supplementary file 1 — Supplementary Information [file 41467_2024_48306_MOESM1_ESM.pdf]

Supplementary information for "Studying phonon coherence with a quantum sensor"

SUPPLEMENTARY NOTE 1: MECHANICS DESIGN

To optimize the mechanical resonator design, we perform finite-element simulations using COMSOL Multiphysics, similar to those detailed in Ref. [11]. Our model includes a small sidewall angle $\theta_{\mathrm{sw}} = 10^\circ$ and corners rounded with radius $r = 80\,\mathrm{nm}$ to account for expected fabrication imperfections. We use X-cut, MgO-doped lithium niobate, with the crystal extraordinary axis oriented perpendicular to the cavity propagation axis. The frequency placement and bandwidth of the phononic bandgap depend on the geometry of the phononic crystal mirror cells. Our choice of design yields a simulated bandgap extending from approximately 1.9 to 2.5 GHz (Supplementary Fig. 1a).

We also perform simulations of the mechanical cavity, which includes the defect site suspended by 4 mirror cells on each side. This is chosen for computational simplicity; the experimental device includes 8.5 mirror cells on each side. The resonant frequency of the localized mode is determined by the defect width, L_x. Target dimensions for both the defect and mirror cells are reported in Supplementary Table 1. From the full structure simulations, we can extract the electromechanical admittance $Y(\omega)$ by simultaneously solving the electrostatic and elastic constitutive relations, coupled by the piezoelectric effect [42]. By applying an oscillating voltage boundary condition at the electrode surface and computing the induced current, we obtain the admittance as the ratio of the two. The simulated results are shown in Supplementary Fig. 1d with a fit to an equivalent circuit model. From this fit, we extract the Butterworth-van Dyke circuit parameters $C_0 = 213.5\,\mathrm{aF}$, $C_{\mathrm{m}} = 51.4\,\mathrm{aF}$, and $L_{\mathrm{m}} = 90.9\,\mu\mathrm{H}$, corresponding to the equivalent circuit shown in the inset.

SUPPLEMENTARY NOTE 2: RAMSEY FIT FUNCTION

Here we discuss the role of mechanical dissipation in fitting the time domain Ramsey data (Eq. 1 of the main text). Though a decay rate proportional to phonon number is theoretically justified for linear decay of a bosonic mode, it is less suitable for our case, as coupling to TLS leads to more complex behavior. In fitting the experimental data, we attempted several approaches, including the original form from Ref. [16] as well as having independent κ_n for each Fock state. We found that for the types of phonon states being analyzed in this study, which have larger average phonon numbers than our previous work, a constant decay of κ assumed for all Fock states yields the best fit quality and stability. We roughly justify this by noting that since TLS are saturated by driving, larger Fock states will tend to have a relatively smaller decay than would be expected from a nonsaturable linear dissipation channel, and so a constant decay across all states is a useful heuristic for fitting the Ramsey signal. Importantly, we only assume this state-independent decay to fit the 700 nanosecond duration of the Ramsey signal, t. The time-dependent phonon occupations are extracted from data over the longer period of measurement, τ.

SUPPLEMENTARY NOTE 3: COHERENT STATE DEPHASING

A harmonic oscillator with resonant frequency ω_0 and annihilation operator $\hat{b}$ can undergo frequency fluctuations due to a stochastic process, $\eta(t)$, leading to a modulated resonant frequency $\omega(t) = \omega_0 + \eta(t)$. In the frame rotating at ω_0, the quantum master equation which describes this process is given by:

$$\frac{d\hat{\rho}}{dt} = \mathcal{D}\big[\frac{1}{\sqrt{T_{2\mathrm{m}}}}\hat{b}^\dagger\hat{b}\big]\hat{\rho} \tag{1}$$

where $\mathcal{D}$ represents the Lindblad dissipator. Classically, these fluctuations of the harmonic oscillator's frequency lead to an equation of motion for a coherent state:

$$\frac{d\alpha}{dt} = -i\eta(t)\alpha \tag{2}$$

which, when integrated, leads to a coherent state amplitude $\alpha(t) = \alpha(0)e^{i\theta(t)}$ where $\theta(t) = \int_0^t \eta(t)dt$. This is reflected in the density matrix as

$$\hat{\rho}(0) = |\alpha\rangle\langle\alpha| \to \hat{\rho}(t) = \sum_\theta p(\theta)|\alpha e^{i\theta}\rangle\langle\alpha e^{i\theta}|. \tag{3}$$

The second displacement operation in the protocol of Fig. 3 of the main text, with amplitude and phase $\alpha e^{i\phi}$, leads to:

$$\hat{D}\hat{\rho}(t)\hat{D}^\dagger = \sum_\theta p(\theta)|\alpha e^{i\theta} + \alpha e^{i\phi}\rangle\langle\alpha e^{i\theta} + \alpha e^{i\phi}|. \tag{4}$$

The resulting phonon number in the oscillator is then given by

$$\begin{aligned}\langle n\rangle &= \sum_\theta p(\theta)|\alpha(e^{i\theta} + e^{i\phi})|^2 \\ &= |\alpha|^2 \sum_\theta p(\theta)(2 + e^{i\phi - i\theta} + e^{i\theta - i\phi}) \\ &= |\alpha|^2\big(2 + e^{i\phi}\langle e^{-i\theta}\rangle + e^{-i\phi}\langle e^{i\theta}\rangle\big)\end{aligned} \tag{5}$$

The expectation values can be expanded as:

$$\langle e^{\pm i\theta}\rangle = \langle 1 \pm i\theta - \frac{\theta^2}{2} + ...\rangle \tag{6}$$

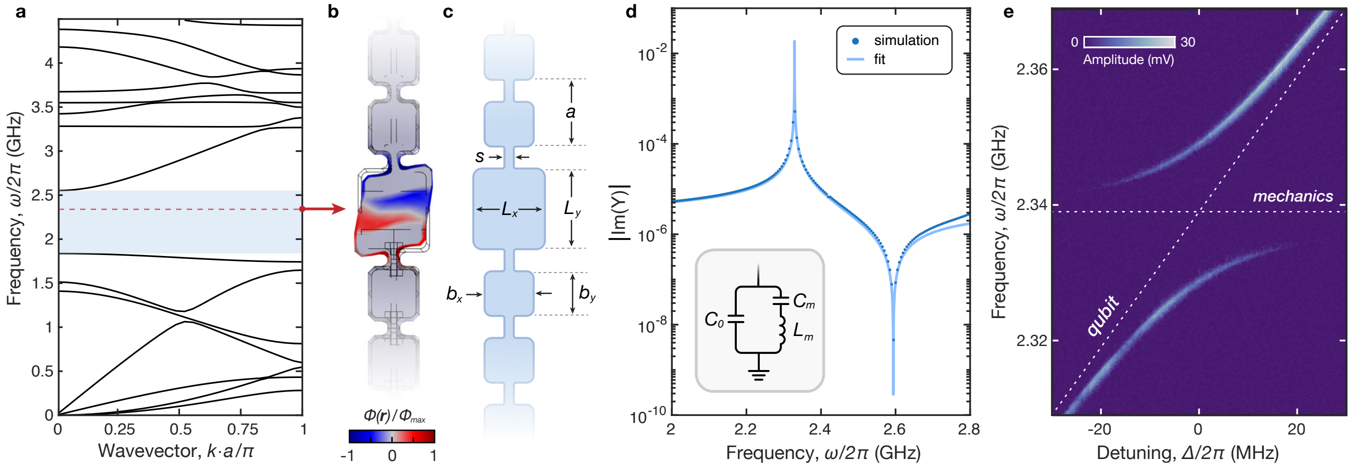

FIG. 1. Supplementary Fig. 1: **Phononic crystal cavity.** **a,** Simulated band structure for the phononic crystal mirror cells, with the primary bandgap highlighted in blue. The red dashed line indicates the measured mechanical frequency from our experimental device, $\omega_\mathrm{m}/2\pi = 2.339\,\mathrm{GHz}$. **b,** Finite element simulation of the mechanical structure including mirror cells, defect site, and electrodes. The localized deformation of the eigenmode is visible, with the color plot indicating the electrostatic potential $\Phi(\vec{r})$. **c,** Important dimensions of the phononic crystal cavity, including both the mirror cells and defect site. Target values for these parameters are reported in Supplementary Table 1. **d,** Simulated admittance $Y(\omega)$ near the mechanical resonance (points) and fit to an equivalent circuit model (line). Inset shows the Butterworth-van Dyke equivalent circuit. The circuit parameters C_0, C_m and L_m extracted from the fit are reported in Supplementary Note and Supplementary Table 1. **e,** Measurement of the qubit excitation spectrum near the mechanical mode. Static flux-tuning of the qubit frequency modifies the detuning $\Delta = \omega_\mathrm{ge} - \omega_\mathrm{m}$ to show an avoided crossing between the two modes.

For a stochastic process $\eta(t)$, the expected value for terms linear in θ are zero: $\langle\theta\rangle = \langle-\theta\rangle = 0$. For short times Δt, the quadratic terms are given by the correlation function $S_{\theta\theta}$:

$$\langle\theta^2\rangle = S_{\theta\theta}(\omega = 0)\Delta t = \frac{2\Delta t}{T_{2\mathrm{m}}} \tag{7}$$

More generally, $\langle\theta^n\rangle = 0$ for odd n, and $\langle\theta^{2n}\rangle \propto (\Delta t)^n$. This allows us to write Eq. 6 in terms of the time increment Δt and decoherence time $T_{2\mathrm{m}}$:

$$\langle e^{\pm i\theta}\rangle = 1 - \frac{\Delta t}{T_{2\mathrm{m}}} + \mathcal{O}(\Delta t)^2 = e^{-t/T_{2\mathrm{m}}} \tag{8}$$

Making this substitution in Eq. 5 brings us to the expected phonon number relation:

$$\langle n\rangle = 2|\alpha|^2\left(1 + e^{-t/T_{2\mathrm{m}}}\cos\phi\right) \tag{9}$$

SUPPLEMENTARY NOTE 4: ESTIMATING MODEL PARAMETERS

The interaction Hamiltonian describing a single TLS coupled to a strain field is given in Refs. [54, 55] as:

$$\hat{H}_\mathrm{int} = \left(\frac{\Delta_\mathrm{as}}{\varepsilon}\hat{\sigma}_z + \frac{\Delta_0}{\varepsilon}\hat{\sigma}_x\right)\gamma\cdot\xi \tag{10}$$

Here, $\hat{\sigma}_x$ and $\hat{\sigma}_z$ are Pauli operators for the TLS, γ is the elastic dipole moment, and ξ is the strain field. Δ_0 and Δ_as represent, respectively, the tunneling energy and asymmetry energy from the bare TLS Hamiltonian, and $\varepsilon = \sqrt{\Delta_0^2 + \Delta_\mathrm{as}^2}$ gives the associated eigenenergies, $\pm\varepsilon/2$. Assuming $\Delta_0/\varepsilon \approx 1$, we can use this relation to estimate the expected coupling rate between the TLS and the strain field, $g_\mathrm{TLS} = \gamma\cdot\xi/\hbar$. The elastic dipole moment can be extracted from material constants and the measured loss tangent for our material platform:[25, 55]

$$\gamma = \sqrt{\delta^0_\mathrm{TLS}\frac{\rho v^2}{\pi P_0}}. \tag{11}$$

Here v is the speed of sound in the host material, ρ the mass density, and P the spectral and spatial density of TLS states. For simplicity, we assume the longitudinal and transverse components of γ to be equivalent, and we use the RMS average of all strain components ξ_{ij} in our calculation.

From a finite-element simulation of the mechanical eigenmode, it is useful to calculate the effective mass

$$m_\mathrm{eff} \equiv \frac{\int dV\rho|u(\vec{r})|^2}{\max|u(\vec{r})|^2} \tag{12}$$

from which we can extract the displacement amplitude $x_\mathrm{zpf} = \sqrt{\hbar/2m_\mathrm{eff}\omega_\mathrm{m}}$, which can be understood as the maximum zero-point fluctuation displacement. For the mode in question, we find $m_\mathrm{eff} = 440\,\mathrm{fg}$ and $x_\mathrm{zpf} = 2.9\,\mathrm{fm}$.

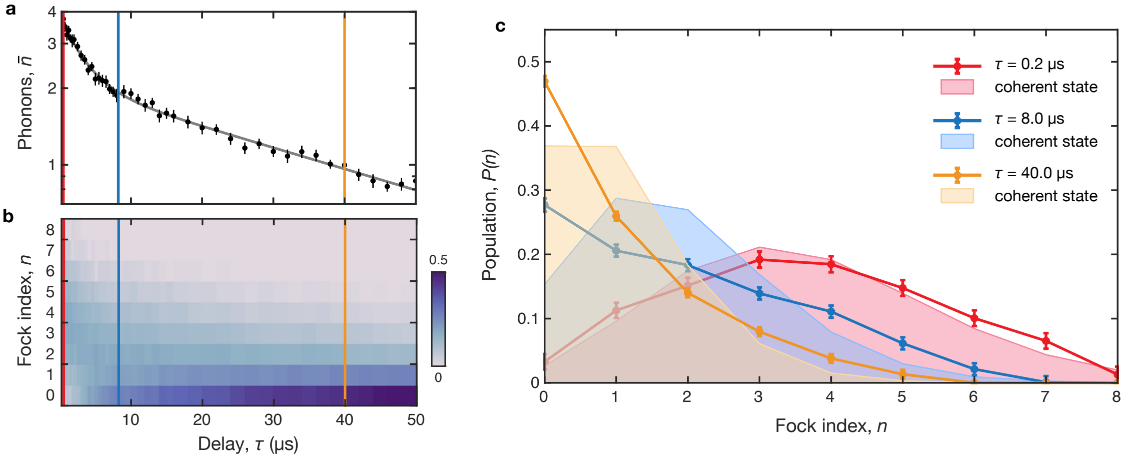

FIG. 2. Supplementary Fig. 2: **Phonon state evolution.** Here we show an expanded view of one ringdown measurement from Fig. 2 of the main text. **a,** Double-exponential ringdown trajectory showing data (points) and fit (line) and **b,** corresponding $P(n)$ evolution, reproduced from Fig. 2d,e. The color bar indicates the population $P(n)$ and vertical lines show the time slices selected for closer examination. **c,** For each selected time slice, we compare the experimentally observed $P(n)$ (points and dark lines) to the coherent state distribution with the same average phonon number (shaded curves). Similar to the effect in Fig. 3f and Fig. 4f of the main text, we observe the measured state distributions diverge from the expected Poissonian form for longer delays.

We can also calculate the average zero-point strain amplitude over the volume of the resonator

$$\bar{\xi}_{\mathrm{zpf}} = \frac{x_{\mathrm{zpf}}}{\max|u(\vec{r})|}\bar{\xi} \tag{13}$$

where $\bar{\xi}$ represents the volume-averaged RMS value of all strain components,

$$\bar{\xi} = \sqrt{\frac{1}{6V}\int dV(\xi_{xx}^2 + \xi_{xy}^2 + \xi_{xz}^2 + \xi_{yy}^2 + \xi_{yz}^2 + \xi_{zz}^2)}. \tag{14}$$

For the phononic crystal resonator mode, we find $\bar{\xi}_{\mathrm{zpf}} = 1.6 \times 10^{-9}$.

The TLS-induced loss tangent at zero temperature δ^0_{TLS} can be determined experimentally. Previous measurements of the temperature-dependent frequency shift of resonators with a similar geometry in this material platform provide an estimated value of the $F\delta^0_{\mathrm{TLS}}$ product [19]. For our system, where we approximate that the TLS are distributed through the entire volume of the cavity, the filling factor is simply $F = 1$.

For the TLS density of states P_0, we assume a value of order $10^{44} - 10^{46}$ $1/\mathrm{J{\cdot}m^3}$ based on literature values extracted for silica whose measured loss tangents are comparable to ours [23, 55]. We calculate the expected distributions for γ and g_{TLS} using Eq. 11 with randomly sampled P_0 and δ^0_{TLS}. For each iteration, we randomly sample $P_0 = 10^{\lambda_1}$ with λ_1 uniformly distributed between $[44, 46]$ and $\delta^0_{\mathrm{TLS}} = 10^{\lambda_2}$ with λ_2 uniformly distributed between $[-4.5, -4]$. We calculate these values using 10,000 iterations to compute the expected distribution for elastic dipole γ and coupling rate g_{TLS}, shown in Supplementary Fig. 3d and 3e.

We can also use P_0 to estimate N, the number of TLS interacting with the mechanical mode. The relevant number to include in our numerical model is the number of TLS within the spatial volume of the resonator V and within a pertinent bandwidth $\delta\omega$ of the mechanical frequency. This bandwidth can be evaluated as $\delta\omega = \max(g_{\mathrm{TLS}}, \gamma_2)$ where γ_2 is the decoherence rate of the TLS. We must also consider the temperature-dependent distribution of the TLS, $P \neq P_0$. For the operating temperature of our experiment ($T = 10\,\mathrm{mK}$), this is found to be $P \simeq 10P_0$ [56]. This allows us to extract

$$N = 10P_0 \times V \times \hbar\delta\omega \tag{15}$$

for a known P_0 and γ_2. The calculations in existing TLS literature predict a much smaller γ_2 arising from TLS-TLS interactions [54]. Our simulation findings are not consistent with this prediction; the model reproduces our experimentally observed effects only in the limit of much faster γ_2. For these values $\gamma_2/2\pi \gtrsim 500$ kHz, Eq. 15 predicts $N \simeq 1 - 5$ TLS. Note that the faster than expected γ_2 is also consistent with the fact that we do not observe coherent oscillations between TLS and the mechanical resonance.

Description	Parameter	Value
pitch	a	900 nm
strut width	s	70 nm
mirror cell width	b_x	575 nm
mirror cell length	b_y	625 nm
defect width	L_x	862 nm
defect length	L_y	1.0 μm
LN thickness		250 nm
Al electrode thickness		50 nm
mBVD coupling capacitance	C_0	213.5 aF
mBVD capacitance	$C_{\rm m}$	51.4 aF
mBVD inductance	$L_{\rm m}$	90.9 μH
LN mass density	ρ	4700 kg/m^3
acoustic wave velocity	v	4000 m/s
TLS density of states	P_0	$10^{45} - 10^{46}$ 1/J·m^3
loss tangent	$\delta^0_{\rm TLS}$	$10^{-4} - 10^{-4.5}$
effective mass	$m_{\rm eff}$	440 fg
zero-point displacement	$x_{\rm zpf}$	2.9 fm
zero-point RMS strain	$\bar{\xi}_{\rm zpf}$	1.6×10^{-9}

TABLE 1. Supplementary Table 1: **Mechanical device parameters.** Physical dimensions, relevant material properties, and extracted parameters corresponding to the mechanical structure, host material, and eigenmode. Target dimensions for the phononic crystal mirror cells and defect site are indicated in Supplementary Fig. 1. Some deviation from these values is expected in the experimental device due to fabrication disorder. Here, mBVD indicates equivalent circuit values from the modified Butterworth-van Dyke model.

SUPPLEMENTARY NOTE 5: ERROR ANALYSIS

We use Monte Carlo error propagation to determine the uncertainties for all values extrapolated from $\bar{n}$ data. First, we extract $P(n)$ from the Ramsey signal $S(t)$ by nonlinear least squares regression, which returns an estimate for the $P(n)$ uncertainties from the fit parameters' covariance matrix. To determine the uncertainty on each calculated phonon number $\bar{n}$, we randomly resample $P(n)$ using the statistical uncertainty in each Fock level population to generate normally-distributed, zero-centered random noise. We repeat this process for 2,000 iterations and calculate $\bar{n}$ at each iteration to build a distribution of values. The reported uncertainty represents the standard deviation of this distribution. The same procedure is applied to determine the uncertainty in extracted values at each level of extrapolation; for fitted values, the resampled data are re-fit at each iteration. Supplementary Fig. 4 shows the distributions generated by this procedure for fitted $T_{\rm 2m}$ in a displacement interferometry experiment as well as κ_1 and κ_2 from a representative nonlinear ringdown measurement.

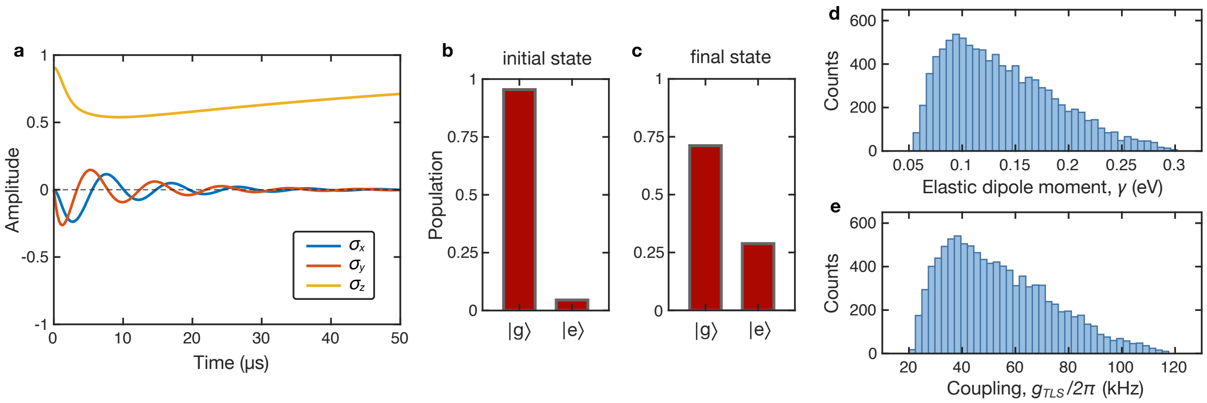

FIG. 3. Supplementary Fig. 3: **TLS state.** **a,** Simulation result showing all Bloch vector components of the TLS state evolution for $g_{\rm TLS}/2\pi = 33\,$kHz, $N = 5$ and $\Delta_{\rm TLS} = 3g_{\rm TLS}$. **b,** Initial TLS state with a small thermal occupation in $|{\rm e}\rangle$. **c,** Final TLS state at the end of the $50\,\mu$s simulation. **d,** Distribution of calculated values for the elastic dipole moment γ and **e,** coupling rate $g_{\rm TLS}$ between the TLS and the strain field. Histograms are constructed from 10,000 iterations.

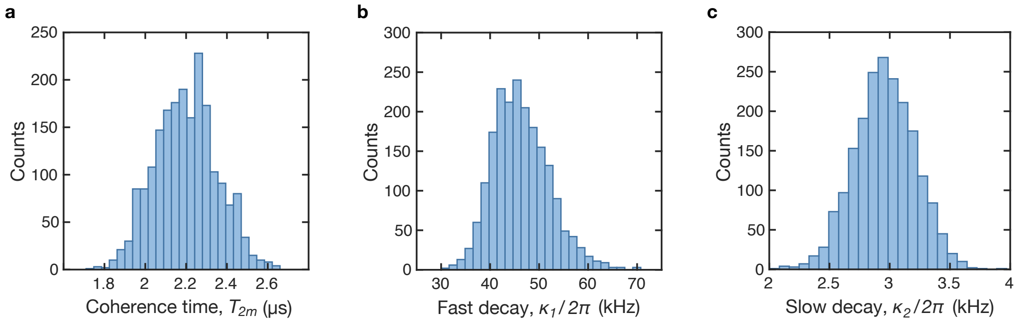

FIG. 4. Supplementary Fig. 4: **Results of uncertainty propagation.** **a,** Distribution of fitted $T_{\rm 2m}$ values for initial state size $\bar{n}_0 = 2.29$. **b,** Fitted values for κ_1 and **c,** κ_2 for a common initial state size $\bar{n}_0 = 2.36$. All reported uncertainties represent the standard deviation of a distribution built from 2,000 repetitions.
